# Editorial

**Published:** 1847-12

**Authors:** 


THE MEDICAL EXAMINER.

PHILADELPHIA, DECEMBER, 1847.

### BRITISH AND FOREIGN MEDICAL REVIEW.

The October number of this admirable Review announces that it is the last which we are to receive. It is melancholy for us to state, that its discontinuance is owing to its never having received such a patronage from the profession as to authorize its distinguished editor to continue it. Still he persevered in his useful exertions until prudence compelled him to abandon the undertaking, and to permit it to pass into other hands, under another form. We have watched this Journal from its inception to its termination. Not a number has escaped our attention; and not a number has been issued that has not afforded us valuable instruction. We are amongst those, who daily feel, more and more, how much we have still to learn, and who mourn over our limited knowledge,—albeit there is no want of effort to maintain ourselves *à portée* with the existing state of science. Doubtless, happy, thrice happy, are they who can admit that "ignorance is bliss;" but this privilege has been denied to those who feel irresistibly, that their life must be spent in incessant toil to allay that thirst for knowledge, which has been implanted in them by the author of nature. To slake this thirst how effectual have been the pure waters issuing, from time to time, from the fount now about to be closed to them. To the profession at large—and especially to the reading, thinking, studying portion—alas! how few in number! the privation of their accustomed intellectual treat from abroad will be felt like the loss of a cherished friend and counsellor; whilst those who are engaged in the active and important occupation of teaching will deplore that one great source of their information, in regard to the novelties and the niceties of science, is about to be shut out from them. May we not hope that the amalgamation of the British and Foreign Medical Review, with its elder brother—the Medico-Chirurgical—may supply the place of the lost one; and that the 'wonted fires' of each parent may burn more brightly in the progeny.

Of Dr. Forbes—who we regret to find is to have no connection with the new Journal—we take our leave with extreme regret; trusting, however, that we shall have constant occasion to witness the continuation of his valuable services to the profession, of which he is so distinguished an ornament. Highly accomplished as a general and professional scholar: dignified and chaste in his language: forcible and fearless in the expression of his opinions: skilled in the selection of reviewers, who were fully competent to their elevated vocation, he was eminently calculated for the lofty editorial position he maintained for the last eleven or twelve years; and although there may be some who writhe under the stripes which he not always mercifully inflicted—and who may not be disposed to kiss the rod that chastened them—we must confess, that in no instance, in our recollection, have we been able to detect unfairness, or to feel that the lashes, although well laid on, were unmerited.

Taking the British and Foreign Medical Review as a whole, we might be justified in indulging the fear, that we may not readily "look upon its like again," seeing, that at the termination of its career, we can state even more fervently than we have done on former occasions, our own conviction that it is—and ever has been—the very best medical review which has appeared in the English language. May we be able to say as much of the "British and Foreign Medico-Chirurgical Review," when it has passed through a long, and we hope glorious course. Whilst we grieve at the loss of its great progenitor, we shall hail its advent with unmixed pleasure.

---

ADULTERATION OF DRUGS.

Constantly have we complaints in the journals of sophistications of many of the most important articles and preparations of the materia medica. The evil has now become so crying, that the College of Pharmacy of New York has decided on petitioning Congress to pass a law, authorizing the appointment of an inspection at the Custom House of imported galenicals and chemicals, and the confiscation of such as are not approved of. In this petition the different medical schools will doubtless unite, as the Jefferson Medical College has already done. We may remark, by the way, that we have recently examined a specimen of the *Pilula Hydrargyri*, prepared by steam power at the chemical laboratory of Mr. George W. Carpenter of this city, which may be depended upon as genuine. It is made in the same manner as at Apothecaries' Hall, London, and the mercury is completely extinguished. This is one of the imported articles which has been most frequently found wanting.

### MEDICAL CLASSES IN PHILADELPHIA.

A month has now elapsed since the lectures commenced in the Medical Schools of Philadelphia, and we are enabled to state that at no former period has the number of students in attendance been greater. Precisely how many are in the city, until the catalogues appear, it is impossible to tell; we have heard the number variously estimated, from a thousand to twelve hundred. Eleven hundred, probably, is not far from correct.

### THE ASIATIC CHOLERA.

The foreign journals continue to furnish the unwelcome intelligence of the march of this pestilence in its old track. At Trebizonde, it appeared on the 9th of September, and continued to increase until the 15th, when the attacks became less numerous, and on the 18th the disease appeared to be on the decline. There had been 300 cases, of which 103 proved fatal. The government medical officer at Trebizonde has remarked that the disease is not so violent as that which formerly devastated Europe, and he adds, that of those who applied to him for advice he saved 90 out of 100. Some cases had been reported in the villages in the neighbourhood of Trebizonde. The disease prevailed both at Bagdad and Imaur-Ali, about the same period, (September.) Late accounts also state that it had appeared at Moscow, and that "Colonel Stalupin, an aid-de-camp of the Emperor of Russia, had fallen a victim to it." The disease had likewise appeared at Odessa. The *Gazette Medicale*, of the 9th of October, informs its readers that "the Cholera has passed the frontiers of Gallicia, and is now spreading through Silesia and Moldavia."

The Dublin Medical Press of the 13th of October, observes: "It is with regret we perceive by announcements in the daily journals that the Asiatic Cholera has reached the interior of Europe, and is gradually extending itself in a north-westerly direction. It is credibly reported that several cases have occurred at Charkav, in south-western Russia, and at Kiev, a large town on the right bank of the Dnieper, and on the frontiers of Poland."

From all the accounts we have seen, it would appear to be certain that the Asiatic Cholera is approaching us by the same route as on the former occasion, and there is great reason to apprehend that it will reach this Continent during the next year. Should such be the case, it is to be hoped that it will not be preceded or accompanied by a similar panic, and that those of the profession who may be obliged to combat it will not desert the established principle of our art, and the evidences of their own senses, to run after every empirical suggestion that may be put forth by ignorance and credulity. It is furthermore to be hoped, that the opportunity will not be allowed to pass, as before, without suitable efforts to establish the pathology of the disease. Chemistry and the miscroscope should be diligently invoked, to reveal the condition of both the solids and the fluids, including all the secretions.

---

NEW YORK ANNALIST.

We regret to find that our well-intended compliment to our brother of the Annalist, on the entrance of his journal upon its *second year*, is deemed by him "*equivocal.*" We assure him that we meant to express no doubtful praise, but unreserved commendation of the zeal and ability displayed in his spirited publication, and a hearty wish for its continued prosperity. A remembrance of the fate of several of his predecessors, and at least one contemporary, in "the Commercial Metropolis," was well calculated to make us rejoice that a better destiny seemed to await him.

